# Mitapivat improves ineffective erythropoiesis and iron overload in adult patients with pyruvate kinase deficiency

**DOI:** 10.1182/bloodadvances.2023011743

**Published:** 2024-02-10

**Authors:** Eduard J. van Beers, Hanny Al-Samkari, Rachael F. Grace, Wilma Barcellini, Andreas Glenthøj, Melissa DiBacco, Megan Wind-Rotolo, Rengyi Xu, Vanessa Beynon, Parija Patel, John B. Porter, Kevin H. M. Kuo

**Affiliations:** 1 Center for Benign Haematology, Thrombosis and Haemostasis, Van Creveldkliniek, University Medical Center Utrecht, Utrecht University, Utrecht, The Netherlands; 2 Division of Hematology, Massachusetts General Hospital, Harvard Medical School, Boston, MA; 3 Dana-Farber/Boston Children’s Cancer and Blood Disorders Center, Harvard Medical School, Boston, MA; 4 Fondazione IRCCS Ca' Granda Ospedale Maggiore Policlinico, Milan, Italy; 5 Danish Red Blood Cell Center, Department of Haematology, Copenhagen University Hospital, Rigshospitalet, Copenhagen, Denmark; 6 Agios Pharmaceuticals, Inc, Cambridge, MA; 7 Haematology Department, University College London Hospitals, London, United Kingdom; 8 Division of Hematology, University of Toronto, Toronto, ON, Canada

## Abstract

Pyruvate kinase (PK) deficiency is a rare, hereditary disease characterized by chronic hemolytic anemia. Iron overload is a common complication regardless of age, genotype, or transfusion history. Mitapivat, an oral, allosteric PK activator, improves anemia and hemolysis in adult patients with PK deficiency. Mitapivat’s impact on iron overload and ineffective erythropoiesis was evaluated in adults with PK deficiency who were not regularly transfused in the phase 3 ACTIVATE trial and long-term extension (LTE) (#NCT03548220/#NCT03853798). Patients in the LTE received mitapivat throughout ACTIVATE/LTE (baseline to week 96; mitapivat-to-mitapivat [M/M] arm) or switched from placebo (baseline to week 24) to mitapivat (week 24 to week 96; placebo-to-mitapivat [P/M] arm). Changes from baseline in markers of iron overload and erythropoiesis were assessed to week 96. Improvements in hepcidin (mean, 4770.0 ng/L; 95% confidence interval [CI], −1532.3 to 11 072.3), erythroferrone (mean, −9834.9 ng/L; 95% CI, −14 328.4 to −5341.3), soluble transferrin receptor (mean, −56.0 nmol/L; 95% CI, −84.8 to −27.2), and erythropoietin (mean, −32.85 IU/L; 95% CI, −54.65 to −11.06) were observed in the M/M arm (n = 40) from baseline to week 24, sustained to week 96. No improvements were observed in the P/M arm (n = 40) to week 24; however, upon transitioning to mitapivat, improvements similar to those observed in the M/M arm were seen. Mean changes from baseline in liver iron concentration by magnetic resonance imaging at week 96 in the M/M arm and the P/M arm were −2.0 mg Fe/g dry weight (dw; 95% CI, −4.8 to −0.8) and −1.8 mg Fe/g dw (95% CI, −4.4 to 0.80), respectively. Mitapivat is the first disease-modifying pharmacotherapy shown to have beneficial effects on iron overload and ineffective erythropoiesis in patients with PK deficiency. This trial was registered at www.ClinicalTrials.gov as #NCT03548220 (ACTIVATE) and #NCT03853798 (LTE).

## Key Points

• Mitapivat was associated with meaningful long-term improvements in key markers of iron homeostasis and erythropoiesis.
• Mitapivat is the first disease-modifying pharmacotherapy to improve iron overload in adult patients with PK deficiency.

## Visual Abstract

**Figure:**
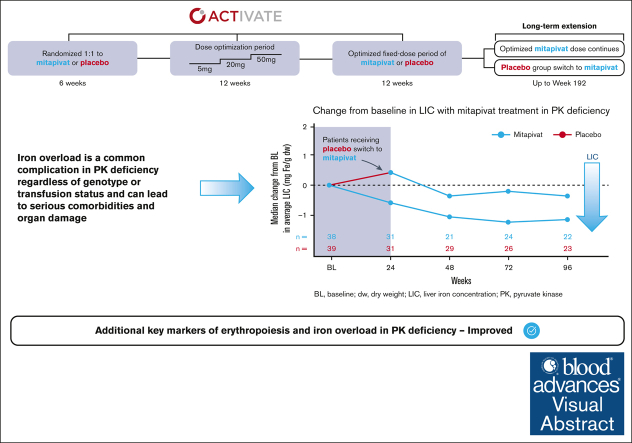

## Introduction

Pyruvate kinase (PK) deficiency is a rare, hereditary disease caused by mutations in the *PKLR* gene that result in chronic hemolytic anemia, ineffective erythropoiesis, and serious complications including iron overload, regardless of age, genotype, or transfusion history.^1^, ^2^, ^3^, ^4^, ^5^, ^6^ Dyserythropoietic features, including key markers associated with iron homeostasis, have been observed in PK deficiency.^7^ Iron overload can lead to increased morbidity^3^^,^^4^ and serious comorbidities including liver cirrhosis, cardiomyopathy, arrhythmia, sudden cardiac death, and endocrine dysfunction.^8^^,^^9^ Iron overload is highly prevalent in patients with PK deficiency regardless of transfusion requirements,^3^, ^4^, ^5^ and its clinical management in PK deficiency is associated with increased morbidity and healthcare costs.^10^^,^^11^ The serious consequences of iron overload further emphasizes the importance of regular monitoring in patients with PK deficiency to prevent additional morbidity and complications. Magnetic resonance imaging (MRI) has emerged as the gold standard for assessment of tissue iron content (such as liver iron concentration [LIC]) because of its accuracy, reproducibility, and noninvasiveness.^12^, ^13^, ^14^

**Figure 1.**
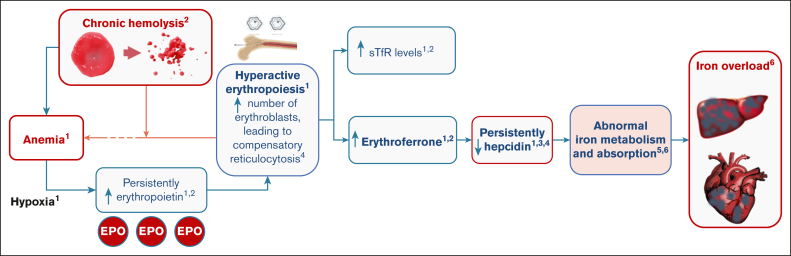
**Pathophysiology of iron overload in PK deficiency.** 1, Adapted from Grootendorst et al^1^; 2, adapted from Zaninoni et al^7^; 3, adapted from van Vuren et al^6^; 4, adapted from Finkenstedt et al^17^; 5, adapted from Gupta et al^18^; 6, adapted from Coffey and Ganz.^19^ EPO, erythropoietin.

In PK deficiency, ineffective erythropoiesis combined with chronic hemolysis can result in iron overload (Figure 1).^7^^,^^15^, ^16^, ^17^, ^18^, ^19^ The erythroferrone–hepcidin axis appears to play a crucial role in the pathogenesis of iron overload. Hypoxia induced by chronic hemolytic anemia drives persistently elevated erythropoietin, which in turn leads to increased erythropoiesis. The increase in erythropoietin induces an increase in erythroferrone production by erythroblasts, which subsequently acts to suppress the production of hepcidin, thereby increasing iron absorption and mobilization to allow for the increased erythropoietic demand.^7^^,^^16^ In an analysis of 115 patients with various rare, hereditary, hemolytic anemias (including PK deficiency), erythropoietin, erythroferrone, and soluble transferrin receptor (sTfR) values were substantially increased in all cohorts compared with healthy controls, and hepcidin values were generally suppressed in all patients with hereditary hemolytic anemias compared with healthy controls.^20^ In another study, it was found that, compared with healthy controls, patients with PK deficiency had higher levels of ineffective erythropoiesis, and increased levels of erythroferrone, erythropoietin, and sTfR.^7^ Patients with PK deficiency also had greater suppression of hepcidin levels than both healthy controls and patients with hereditary spherocytosis, and had dyserythropoietic features independent of their transfusion status.^7^^,^^21^

Mitapivat is a first-in-class, oral, allosteric activator of red blood cell wild-type and mutant PK enzyme that is approved by the US Food and Drug Administration for the treatment of hemolytic anemia in adults with PK deficiency^22^ and by the European Medicines Agency in the European Union and the Medicines and Healthcare Products Regulatory Agency in Great Britain for the treatment of PK deficiency in adult patients.^23^^,^^24^ Mitapivat has demonstrated improvements in hemoglobin, hemolysis, and transfusion burden in 2 global, phase 3 trials in adult patients with PK deficiency who were either not regularly transfused (ACTIVATE; www.clinicaltrials.gov identifier: NCT03548220)^25^ or who were regularly transfused (ACTIVATE-T; www.clinicaltrials.gov identifier: NCT03559699).^26^ In ACTIVATE, 16 of 40 (40%) patients on mitapivat achieved hemoglobin response (defined as ≥1.5 g/dL increase in hemoglobin concentration from baseline, sustained at ≥2 scheduled assessments at weeks 16, 20, and 24 during the fixed-dose period) compared with 0 of 40 (0%) patients on placebo (2-sided *P* < .0001).^25^ Patients receiving mitapivat also demonstrated greater improvements in markers of hemolysis (indirect bilirubin, lactose dehydrogenase, and haptoglobin levels) than those receiving placebo, and experienced significant improvements compared with placebo in 2 PK deficiency–specific patient-reported outcomes that assessed the signs, symptoms, and impact of PK deficiency.^25^ In addition, mitapivat was well tolerated, with a safety profile consistent with previous studies.^25^^,^^27^

This manuscript presents data from the ACTIVATE clinical trial and its long-term extension (LTE) study (NCT03853798) and evaluates the impact of mitapivat on iron overload and ineffective erythropoiesis.

## Methods

**Figure 2.**
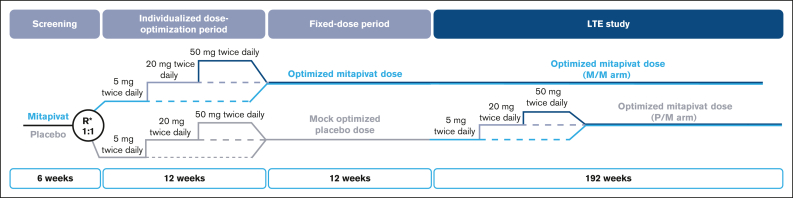
**ACTIVATE/LTE study design.** Key eligibility criteria: (1) age of ≥18 years; (2) documented 2 mutant alleles in *PKLR* with 1 missense mutation (excluding patients homozygous for R479H mutation or who have 2 nonmissense mutations, without another missense mutation); (3) ACTIVATE: not regularly transfused (≤4 transfusion episodes in the previous year); baseline hemoglobin of ≤10 g/dL; (4) LTE study: completed the fixed-dose period of ACTIVATE and demonstrated clinical benefit from mitapivat treatment, or were assigned to the placebo arm in ACTIVATE and elected to continue to the LTE study. ∗Stratified by average of screening hemoglobin values (<8.5 g/dL vs ≥8.5 g/dL) and *PKLR* gene mutation category (missense/missense vs missense/nonmissense). BID, twice daily; R, randomized.

ACTIVATE was a global, phase 3, double-blind, placebo-controlled study of mitapivat in adults with PK deficiency who were not regularly transfused (≤4 transfusion episodes in the prior year).^25^ In ACTIVATE, patients received mitapivat or placebo for 24 weeks. In the 12-week dose-optimization period, mitapivat or placebo was administered at an initial oral dosage of 5 mg twice daily. Two potential sequential steps at weeks 4 and 8 were permitted to increase the dose from 5 mg to 20 mg, and then from 20 mg to 50 mg, based on safety and efficacy assessments. Doses at week 12 were then maintained through the following 12-week fixed-dose period. Patients who, in the opinion of the investigator, demonstrated a clinical benefit from mitapivat upon completion of the fixed-dose period of ACTIVATE (from weeks 12 to 24) or who were assigned to the placebo arm in ACTIVATE were eligible to continue in the LTE. All patients enrolled in the LTE received mitapivat in either the mitapivat-to-mitapivat (M/M) arm or the placebo-to-mitapivat (P/M) arm. Patients in the M/M arm maintained their optimized treatment dosage from ACTIVATE, whereas patients in the P/M arm received mitapivat at an initial dosage of 5 mg twice daily and could sequentially increase their dose to 20 mg or 50 mg, as in ACTIVATE (Figure 2). The trial protocol was approved by an institutional review board or independent ethics committee at each participating institution, and all the patients provided written informed consent.

In this analysis, changes from baseline in key markers of iron overload and erythropoiesis were assessed up to week 96 for both study arms (M/M and P/M), including hepcidin, erythroferrone, sTfR, LIC by MRI, ferritin, erythropoietin, and reticulocyte percentage. The change in LIC by MRI was also analyzed in a subset of patients considered to have iron overload at baseline, which was defined as the presence of any 1 or more of the following 3 criteria at baseline: ferritin of >1000 μg/L, LIC of >3 mg Fe/g dry weight (dw), and/or any treatment with chelation therapy within the last year before start of treatment with mitapivat, regardless of the number or duration of chelation treatments.^4^ A 1.5 Tesla R2 MRI was used. LIC data from the M/M and P/M arms were pooled and summarized to week 96. The baseline for LIC by MRI was defined as the last assessment before randomization for patients randomized and not dosed, or the last assessment before the start of study treatment for patients randomized and dosed. The baseline for other assessments was defined as the average of all screening assessments before the start of study treatment. Assessments collected within 61 days after a transfusion were excluded from the baseline derivation. Patients in the M/M arm started mitapivat treatment at baseline; patients in the P/M arm started mitapivat treatment at week 24.

The status of chelation therapy while on mitapivat treatment was also described. For those on chelation, the number and proportion of patients who discontinued chelation treatment, increased or decreased their chelation dose, or remained on a stable chelation dose while on treatment with mitapivat were reported.

No formal statistics were used; data were summarized descriptively. Categorical variables were summarized by number and percentage. Continuous variables were summarized using either mean and standard deviation (SD) or mean and 95% confidence intervals (CIs), with the exception of LIC by MRI for which median and quartile (Q) 1 and Q3 were also reported to account for 2 incorrectly reported values above the reportable range at baseline and week 24.

## Results

### Baseline characteristics

A total of 80 patients were included in the ACTIVATE/LTE analysis (M/M = 40; P/M = 40); baseline characteristics for patients in ACTIVATE have been reported previously.^25^ Patients enrolled in ACTIVATE had a high disease burden at baseline, with clinical markers indicative of chronic hemolytic anemia and iron overload, and a high incidence of splenectomy and cholecystectomy (both ≥70%).^25^ In brief, mean (SD) hemoglobin levels were 8.6 g/dL (1.0) and 8.5 g/dL (0.8) in the M/M and P/M arms, respectively; indirect bilirubin was 81.8 μmol/L (61.3) and 89.1 μmol/L (61.8), and reticulocyte percentage was 37.1% (24.1) and 40.1% (22.2), respectively. Patients in both arms had abnormal baseline levels of biomarkers and clinical parameters of iron overload.^5^^,^^25^ In the M/M and P/M arms, mean (SD) hepcidin was 25 920 ng/L (27 900) and 29 989 ng/L (18 044), erythroferrone was 21 080 ng/L (16 029) and 20 380 ng/L (13 096), ferritin was 748 μg/L (1116) and 688 μg/L (605), and sTfR was 187 nmol/L (76) and 174 nmol/L (69), respectively; median (Q1, Q3) LIC by MRI was 3.05 mg Fe/g dw (1.70, 6.50) and 3.40 mg Fe/g dw (2.00, 6.30), with mean (SD) LIC by MRI 7.6 mg Fe/g dw (10.8) and 6.1 mg Fe/g dw (8.0), respectively (Table 1).

**Table 1.** **Change from baseline in markers of iron metabolism and iron overload at week 24 and week 96 in patients with PK deficiency in ACTIVATE and the LTE study**

Marker	All patients					
	Baseline		Change from baseline at week 24		Change from baseline at week 96	
	M/M N = 40	P/M N = 40	M/M N = 40	P/M N = 40	M/M N = 40	P/M N = 40
Hepcidin, mean (SD) (95% CI), ng/L∗	25 920 (27 900) n = 40	29 989 (18 044) n = 40	4770 (18 347) (−1532 to 11 072) n = 35	−3282 (14 735) (−8687 to 2123) n = 31	6009 (23 135) (−2791 to 14 809) n = 29	9935 (23 580) (−262 to 20 132) n = 23
Erythroferrone, mean (SD) (95% CI), ng/L∗	21 080 (16 029) n = 40	20 380 (13 096) n = 40	−9835 (13 081) (−14 328 to −5 341) n = 35	−2133 (6 278) (−4 436 to 170) n = 31	−12 635 (13 019) (−17 587 to 7 683) n = 29	−10 720 (6729) (−13 630 to −7810) n = 23
sTfR, mean (SD) (95% CI), nmol/L∗	187 (76) n = 40	174 (69) n = 40	−56 (83) (−85 to −27) n = 34	−2 (17) (−9 to 5) n = 28	−60 (85) (−93 to −27) n = 28	−31 (41) (−48 to −13) n = 25
Erythropoietin, mean (SD) (95% CI), IU/L∗	74 (60) n = 39	74 (57) n = 40	−33 (62) (−55 to −11) n = 34	7 (38) (−7 to 21) n = 30	−32 (71) (−59 to −4) n = 28	−31 (54) (−53 to −9) n = 26
Reticulocyte percentage, mean (SD) (95% CI), %∗	37.1 (24.1) n = 40	40.1 (22.2) n = 40	−10.7 (10.9) (−14.5 to −7.0) n = 35	−0.4 (9.9) (−3.9 to 3.0) n = 34	−9.6 (11.6) (−14.5 to −4.7) n = 24	−9.5 (14.6) (−15.6 to −3.5) n = 25
Ferritin, mean (SD) (95% CI), μg/L∗	748 (1116) n = 39	688 (605) n = 38	39 (285) (−57 to 136) n = 36	−50 (217) (−130 to 29) n = 31	25 (188) (−48 to 98) n = 28	59 (393) (−100 to 218) n = 26
LIC assessment by MRI, mg Fe/g dw†	n = 38	n = 39	n = 31	n = 31	n = 22	n = 23
Mean (SD) (95% CI)	7.6 (10.8)	6.1 (8.0)	1.7 (15.7) (−4.1 to 7.5)	1.4 (12.4) (−3.2 to 5.9)	−2.0 (6.2) (−4.8 to 0.8)	−1.8 (6.0) (−4.4 to 0.8)
Median (Q1, Q3)‡	3.05 (1.70, 6.50)	3.40 (2.00, 6.30)	−0.40 (−1.10, 0.70)	0.30 (−0.30, 1.20)	−0.85 (−1.90, −0.10)	−0.30 (−1.30, 0.70)

	Patients with iron overload at baseline who received mitapivat§ N = 43		
	Baseline	Change from baseline at week 24	Change from baseline at week 96
LIC assessment by MRI, mg Fe/g dw†	n = 43	n = 37	n = 20
Mean (SD) (95% CI)	11.7 (13.1)	−0.8 (18.2) (−6.9 to 5.3)	−3.3 (6.6) (−6.4 to −0.3)
Median (Q1, Q3)‡	6.50 (4.10, 15.60)	−0.90 (−2.50, 0.40)	−1.95 (−4.85, −0.70)

Patients in the M/M arm started mitapivat treatment at baseline; patients in the P/M arm started mitapivat treatment at week 24.

95% CI was calculated based on t-distribution.

∗ The baseline is defined as the average of all screening assessments within 45 (42 + 3) days before randomization for patients randomized and not dosed, or before the start of study treatment for patients randomized and dosed. Assessments collected within 61 days after a transfusion were excluded from the baseline derivation.

† The baseline for LIC by MRI is defined as the last assessment before randomization for patients randomized and not dosed, or the last assessment before the start of study treatment for patients randomized and dosed.

‡ Median (Q1, Q3) was reported for LIC by MRI in addition to mean (SD) [95% CI] because of 2 incorrectly reported values, which were included in the analysis. These 2 outlier values for LIC limit the interpretation of the summaries of mean change in LIC from baseline to week 24; data for mean change from baseline to week 96 were not affected.

§ Patients were considered to have iron overload at baseline if they met at least 1 of 3 criteria: baseline ferritin of >1000 μg/L, baseline LIC of >3 mg Fe/g dw, and/or chelation therapy within the last year before start of treatment with mitapivat.

Of 80 patients included in the ACTIVATE/LTE analysis, 78 were treated with mitapivat (1 patient was randomized to placebo in ACTIVATE but discontinued from the study before receiving treatment [reason: lost to follow-up]; 1 patient randomized to placebo in ACTIVATE did not continue into the LTE). Of the 78 patients treated with mitapivat and enrolled in the study, 22 (28.2%) had both LIC of >3 mg Fe/g dw and ferritin of <1000 μg/L at baseline, and 13 (16.7%) had LIC of >5 mg Fe/g dw and ferritin of <1000 μg/L, further demonstrating that iron overload occurs in patients with PK deficiency regardless of transfusions. Of 78 patients treated with mitapivat, 43 (55.1%) met the criteria for iron overload at baseline (Table 2), with a median (Q1, Q3) and a mean (SD) LIC by MRI of 6.50 mg Fe/g dw (4.10, 15.60) and 11.7 (13.1), respectively, in this patient subgroup (Table 1).

**Table 2.** **Key baseline characteristics related to iron overload**

Criteria, n (%)∗	Mitapivat N = 78
**Baseline ferritin of >1000 μg/L**†	
Yes	15 (19.2)
No	61 (78.2)
Missing	2 (2.6)
**Baseline average LIC by MRI of >3 mg Fe/g dw**‡	
Yes	39 (50.0)
No	37 (47.4)
Missing	2 (2.6)
**Prior chelation status**§	
Yes	16 (20.5)
No	62 (79.5)
**Iron overload at baseline**||	
Yes	43 (55.1)
No	35 (44.9)

∗ The denominator used to calculate percentages is N, the number of subjects in the full analysis set.

† Ferritin values reported as >1500 μg/L because of a laboratory dilution error were excluded; the baseline for ferritin is the average of all assessments within 45 (42 + 3) days before the start of treatment with mitapivat; assessments collected within 61 days after a transfusion were excluded from the baseline derivation.

‡ The baseline for average LIC by MRI is defined as the last assessment before the start of treatment with mitapivat.

§ Prior chelation status was established as part of medical history to distinguish from assessment of chelation on treatment; “Yes” if a subject had received chelation therapy within 52 weeks (364 days) before the start of treatment with mitapivat.

|| “Yes” if subject met at least 1 of 3 criteria: baseline ferritin of >1000 μg/L, baseline average LIC of >3 mg Fe/g dw, and/or prior chelation status = Yes.

### Change from baseline in markers of iron overload and erythropoiesis

Directional improvements in markers of iron homeostasis and erythropoiesis that are associated with iron overload (hepcidin, erythroferrone, sTfR, erythropoietin, and reticulocyte percentage, as well as LIC by MRI) were observed in patients treated with mitapivat from baseline to week 24 in the M/M arm and were sustained from week 24 to week 96 in the LTE (Table 1). In patients treated with placebo in ACTIVATE, these markers remained relatively unchanged from baseline to week 24, but improvements similar to those observed in the M/M arm were observed from week 24 to week 96 upon transition to mitapivat in the LTE.

### Change from baseline in hepcidin

**Figure 3.**
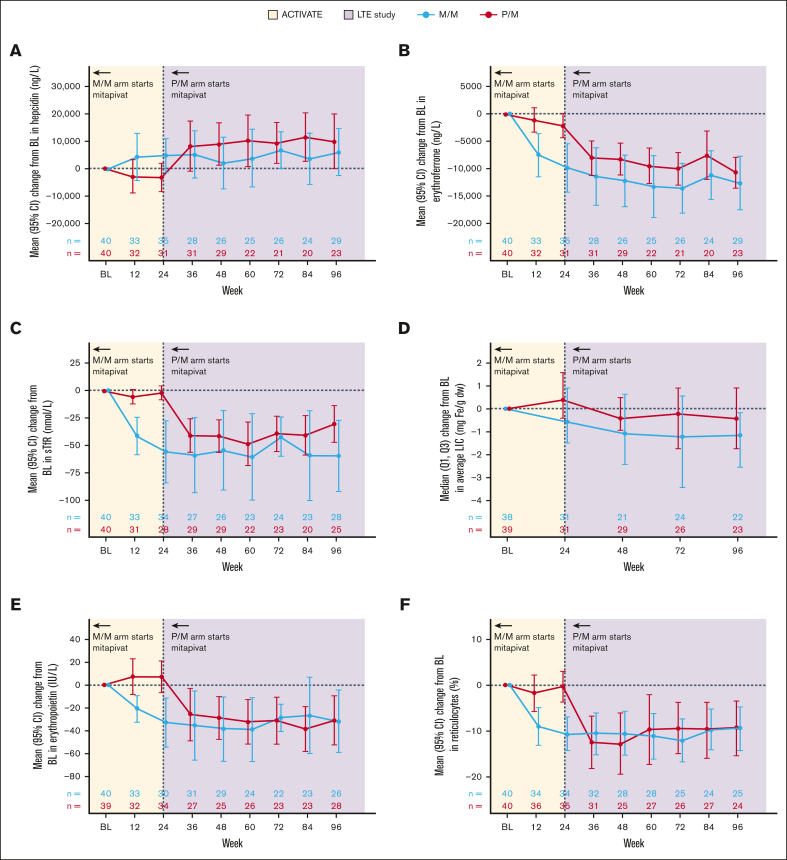
**Changes from baseline in markers of iron homeostasis with treatment with mitapivat in the ACTIVATE/LTE trial.** (A) Hepcidin, (B) erythroferrone, (C) sTfR, (D) LIC by MRI, (E) erythropoietin, and (F) reticulocyte percentage. The baseline for LIC by MRI is defined as the last assessment before randomization for patients randomized and not dosed, or the last assessment before the start of study treatment for patients randomized and dosed. The baseline for the other parameters is defined as the average of all screening assessments within 45 (42 + 3) days before randomization for patients randomized and not dosed, or before the start of study treatment for patients randomized and dosed. Assessments collected within 61 days after a transfusion were excluded from the baseline derivation. n is the number of patients in the full analysis set within each treatment group who had an assessment at the visit or who (for the summaries of change from baseline) had a baseline assessment and ≥1 postbaseline assessment at the visit. 95% CI was calculated based on t-distribution. BL, baseline.

In the M/M arm, directional improvements in hepcidin were observed, with increases from baseline to week 24 (mean change from baseline, 4770 ng/L; 95% CI, −1532 to 11 072) and week 96 (mean change from baseline, 6009 ng/L; 95% CI, −2791 to 14 809). Hepcidin levels worsened slightly while patients received placebo in the P/M arm from baseline to week 24 (mean change from baseline, −3282 ng/L; 95% CI, −8687 to 2123), but similar increases to the M/M arm were observed from week 24 to week 96 after mitapivat treatment in the LTE (mean change from baseline to week 96, 9935 ng/L; 95% CI, −262 to 20 132; Figure 3A; Table 1).

### Change from baseline in erythroferrone and sTfR

Directional improvements were observed in erythroferrone levels, which decreased from baseline to weeks 24 and 96 after mitapivat treatment in the M/M arm (mean change from baseline to week 24, −9835 ng/L [95% CI, −14 328 to −5341]; mean change from baseline to week 96, 12 635 ng/L [95% CI, −17 587 to −7683]). Similar decreases were observed in patients in the P/M arm from week 24 to week 96. sTfR levels were also decreased in the M/M arm at weeks 24 and 96, with similar reductions observed from week 24 to week 96 in the P/M arm (Figure 3B-C; Table 1).

### Change from baseline in erythropoietin

Erythropoietin levels decreased in the M/M arm from baseline to weeks 24 and 96 (mean change from baseline to week 24, −33 IU/L [95% CI, −55 to −11]; and to week 96: −32 IU/L [95% CI, −59 to −4]), with similar directional changes from week 24 to week 96 in the P/M arm (Figure 3E; Table 1).

### Change from baseline in reticulocyte percentage

Decreases in reticulocyte percentage were observed with mitapivat in the M/M arm from baseline to weeks 24 and 96, and similar results were seen in the P/M arm from week 24 to week 96 after the start of mitapivat treatment (Figure 3F; Table 1).

### Change from baseline in ferritin

Ferritin remained stable in both the M/M and P/M groups from baseline to week 96. In the M/M group, the mean change from baseline to week 24 was 39 μg/L (95% CI, −57 to 136) and to week 96 was 25 μg/L (95% CI, −48 to 98). In the P/M group, the mean change to week 24 was −50 μg/L (95% CI, −130 to 29), and to week 96 was 59 μg/L (95% CI, −100 to 218; Table 1).

### Change from baseline in LIC by MRI

Median (Q1, Q3) data were reported for LIC by MRI because of the inclusion of 2 incorrectly reported values in the analysis which were above the reportable range; these values limit the interpretation of the summaries of mean change in LIC by MRI from baseline at week 24 (data for mean change from baseline at week 96 were not affected). LIC by MRI decreased from baseline to weeks 24 and 96 in the M/M arm, with median changes of −0.40 mg Fe/g dw (Q1, Q3: −1.10, 0.70) and −0.85 mg Fe/g dw (−1.90, −0.10), respectively, and mean changes of 1.7 mg Fe/g dw (95% CI, −4.1 to 7.5) and −2.0 mg Fe/g dw (95% CI, −4.8 to 0.8), respectively. LIC by MRI showed no improvement from baseline to week 24 in the P/M arm (median change, 0.30 mg Fe/g dw [Q1, Q3: −0.30, 1.20]; mean change, 1.4 mg Fe/g dw [95% CI, −3.2 to 5.9]), which was followed by a decrease from week 24 to week 96 after the transition to mitapivat in the LTE (median [Q1, Q3] change from baseline to week 96: −0.30 mg Fe/g dw [−1.30, 0.70]; mean change, −1.8 mg Fe/g dw [95% CI, −4.4 to 0.8]; Figure 3D; Table 1).

**Figure 4.**
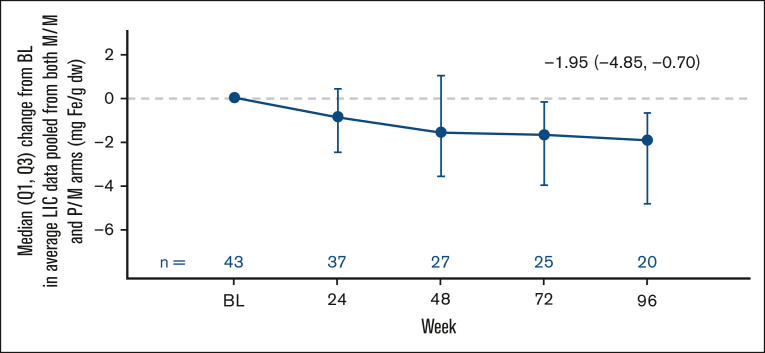
**Pooled change from baseline in LIC after treatment with mitapivat in patients with baseline iron overload.** Patients were considered to have iron overload at baseline if they met at least 1 of 3 criteria: baseline ferritin of >1000 μg/L, baseline LIC of >3 mg Fe/g dw, and/or chelation therapy within the last year before start of treatment with mitapivat. The baseline for LIC by MRI is defined as the last assessment before randomization for patients randomized and not dosed, or the last assessment before the start of study treatment for patients randomized and dosed. BL, baseline.

Furthermore, change from baseline in LIC by MRI was also evaluated in the subgroup of patients treated with mitapivat with iron overload at baseline (43/78 [55.1%]; median LIC at baseline: 6.50 mg Fe/g dw [Q1, Q3: 4.10, 15.60]; mean at baseline 11.7 mg Fe/g dw [SD, 13.1]). Mitapivat treatment led to meaningful and sustained improvements in LIC by MRI (median change from baseline to week 24, −0.90 mg Fe/g dw [Q1, Q3: −2.50, 0.40]; and to week 96, −1.95 mg Fe/g dw [−4.85, −0.70]; mean change from baseline to week 24, −0.8 mg Fe/g dw [95% CI, −6.9 to 5.3]; and to week 96, −3.3 mg Fe/g dw [95% CI, −6.4 to −0.3]; Figure 4; Table 1).

### Chelation

Overall, the majority of patients (57/78 [73.1%]) in the trial were not receiving chelation at baseline (defined as commencement of mitapivat treatment) and did not receive chelation while on treatment. Of 21 patients who were on chelation while receiving mitapivat, 7 of 21 (33.3%) discontinued chelation; 4 of 21 (19.0%) decreased their chelation dose relative to that at baseline; 6 of 21 (28.6%) had an increase in their chelation dose relative to that at baseline, or started new chelation therapy while on treatment; and 4 of 21 (19.0%) remained on a stable dosage.

## Discussion

Analysis of the ACTIVATE phase 3 clinical trial and LTE data showed that PK activation with mitapivat led to long-term improvements in key systemic regulators of iron homeostasis and iron overload in patients with PK deficiency. Notably, patients in the P/M arm showed improvements upon transitioning to mitapivat in the LTE that were consistent with the initial improvements seen in the mitapivat-treated group (M/M arm) in the ACTIVATE trial. Together, these changes demonstrate an improvement in iron burden with mitapivat treatment, which was confirmed through the assessment of LIC by MRI. Ferritin levels were stable from baseline to week 96 after mitapivat treatment. However, from data presented both here and in the PK deficiency Natural History Study, ferritin appears to underestimate the severity of iron overload in hereditary hemolytic anemias when compared with LIC determined by MRI and thus represents a poor predictor of systemic iron burden, even when patients have moderate to severe iron overload.^4^ This may be because of the high erythropoietic drive causing severe hepcidin deficiency, which, in turn, leads to iron depletion of macrophages and lowering of the secretion of ferritin.^28^, ^29^, ^30^ The incongruency between LIC and ferritin levels at baseline further highlights the high prevalence of iron overload even in patients who are not regularly transfused, and the importance of iron screening in all patients with PK deficiency.^4^

Hemolytic anemia is associated with persistent elevation of sTfR and erythroferrone, which is engaged as a compensatory mechanism to attempt to alleviate the anemic state through erythrocyte proliferation^17^^,^^31^^,^^32^ and the suppression of hepcidin activity to increase the uptake of the iron normally required to support erythropoiesis.^7^^,^^33^ However, when serum iron concentration is already sufficient for proliferative demand, this chronic signaling leads to a pathologic increase in iron absorption, resulting in iron overload.^15^ Patients treated with mitapivat showed decreases in both sTfR and erythroferrone, increases in hepcidin, and improvements in LIC from baseline, with improvements in LIC particularly marked in the subgroup of patients with iron overload at baseline. This is of particular importance, given the serious consequences and additional disease burden that these patients face.^3^^,^^4^^,^^8^^,^^9^ Overall, mitapivat treatment was associated with alleviation of iron overload. This process is likely mediated by an improvement in erythropoiesis via indirect modulation of the erythroferrone–hepcidin axis, with potential contribution from the changes in hemolysis that also occur after mitapivat treatment.^34^ Moreover, the results presented here further highlight the importance of the assessment of LIC by MRI. As the gold standard for the assessment of liver iron burden in patients with PK deficiency, this method ensures that iron burden is appropriately assessed and that patients receive optimal management.^4^

The pathologic features of iron overload in patients have been established and are known to result in numerous serious additional complications,^8^^,^^9^ and there is substantial cost and morbidity associated with the therapeutic management of iron overload.^11^ The improvements in iron overload observed after the mitapivat-mediated reduction of hemolytic anemia and improvements in ineffective erythropoiesis in these patients may therefore have the potential to reduce both the morbidity and the economic burden associated with iron overload and its management in patients with PK deficiency regardless of transfusion status.^3^^,^^4^^,^^10^

The limitations of the study include that the population in the analysis comprised adult patients with PK deficiency who were not regularly transfused; thus, the findings presented may not be generalizable to the full spectrum of patients with PK deficiency. Furthermore, this was an exploratory post hoc analysis and therefore it was descriptive in nature, with no formal statistics used.

In conclusion, treatment with mitapivat showed clinically meaningful and durable improvements in LIC by MRI, demonstrating its impact on total body iron burden in PK deficiency. Changes in key systemic regulators of iron homeostasis and markers of erythropoietic activity are also in keeping with the change in systemic iron burden with long-term mitapivat treatment. Mitapivat is the first disease-modifying pharmacotherapy shown to have beneficial effects on iron overload in adult patients with PK deficiency through its multimodal action, including modulating the erythroferrone–hepcidin axis.

Conflict-of-interest disclosure: E.J.v.B. is a member of the advisory board for Agios and receives research funding from Agios, Novartis, Pfizer, and RR Mechatronics. H.A.-S. is a consultant for Agios, Argenx, Forma, Moderna, Novartis, Rigel, Sobi, and Pharmacosmos, and receives research funds from Agios, Amgen, Sobi, Novartis, and Vaderis. R.F.G. receives research funds from Agios, Novartis, and Sobi, and is a consultant for Agios and Sanofi. W.B. receives honoraria from Agios, Alexion, and Novartis; received research funds from Agios; and is a board member and advisory committee member for Bioverativ and Incyte. A.G. is a consultant and a member of the advisory board for Agios, bluebird bio, Bristol Myers Squibb, Novartis, Novo Nordisk, and Pharmacosmos, and receives research support from Agios, Saniona, and Sanofi. M.D., M.W.-R., R.X., V.B., and P.P. are all employees and shareholders of Agios. J.B.P. receives honoraria from Agios, bluebird bio, Celgene, La Jolla Pharmaceuticals, Protagonism, Silence Therapeutics, and Vifor, and is a consultant for Agios, bluebird bio, and Celgene. K.H.M.K. is a consultant for Agios, Alexion, Apellis, bluebird bio, Celgene, Novartis, and Pfizer; receives honoraria from Alexion and Novartis; is a member of an entity’s board of directors and advisory committees for Agios and Bioverativ/Sanofi/Sangamo; and received research funding from Agios and Pfizer.
